# Intra-unit reliability and movement variability of submission grappling external load as measured by torso mounted accelerometery

**DOI:** 10.5114/biolsport.2023.114287

**Published:** 2022-06-01

**Authors:** Christopher Kirk, James J Malone, Peter J Angell

**Affiliations:** 1 Sport and Human Performance Research Group, Sheffield Hallam University, Collegiate Crescent, Sheffield, S10 2NA; 2 Research Institute for Sport and Exercise Sciences, Liverpool John Moores University, Byrom St., Liverpool, L3 5AF; 3 School of Health Sciences, Liverpool Hope University, Hope Park, Liverpool, L16 9JD

**Keywords:** PlayerLoad, Combat sports, Training load, Grappling, Athlete monitoring

## Abstract

Submission grappling consists of skills and movements used in combat sports to physically control opponents whilst trying to apply choke holds and joint locks. There is currently no accepted method of monitoring external load in grappling-based sports due to the absence of key variables such as distance, velocity or time. The primary aim of this study was to determine whether PlayerLoad is a reliable variable for measuring external load of submission grappling movements, with a secondary aim of determining the between repetition variance of submission grappling movements. 7 experienced submission grapplers were recruited. Each wore a torso mounted Catapult^®^ Optimeye S5 microelectromechanical systems (MEMS) device and completed 5 repetitions of each of the following: 4 submission techniques; 5 transition techniques; 2 guard pass techniques; 2 takedown techniques. Accumulated PlayerLoad (PLd_ACC_) was recorded as a marker of absolute load, with accumulated PlayerLoad per minute (PLd_ACC_∙min^-1^) representing relative load. Reliability of each was assessed using intraclass correlation coefficient (ICC_(3,1)_) (≥ 0.70). Between repetition movement variation was assessed via coefficient of variation with 95% confidence intervals (CV, 95%CI) (acceptable ≤ 15%, good ≤ 10%). PLd_ACC_ ICC_(3,1)_ range = 0.78–0.98, with CV range = 9–22%. PLd_ACC_∙min^-1^ ICC_(3,1)_ range = 0.83–0.98, with CV range = 11–19%. Though several variables displayed CV > 15%, all had 95%CI lower boundaries ≤ 15%. Whilst PlayerLoad was found to be a reliable measure for submission grappling, relatively high CVs across most techniques examined suggest PlayerLoad may not be appropriate for measuring changes in external load for individual movements in submission grappling. However, it may prove a useful tool for monitoring the external load of full, grappling-based, training sessions within an individual.

## INTRODUCTION

Submission grappling refers to a series of skills, techniques and movements used in a range of combat sports including: Brazilian jiu jitsu (BJJ), mixed martial arts (MMA), sambo, wrestling and judo [1]. The aim is for participants to attain and maintain a dominant position over an opponent in order to apply joint manipulations or choke holds to make them admit defeat by ‘submitting’ [2]. While submission grappling techniques are a secondary skill in wrestling, judo and sambo [3–5], they are a key skill component in both MMA and BJJ respectively [6, 7]. As with most sports, understanding and quantifying the physiological load imposed during grappling based training, sparring and competition is important in order to manipulate and manage training loads effectively [8].

As previous work has described, the outcome of a training process is dependent on the external and internal training load an individual is exposed to. External load refers to the physical actions completed in a training session, whereas internal load refers to the physiological response of the individual to the imposed external load [8]. Due to the combative nature of submission grappling, there are inherent difficulties in directly measuring internal and external loads of participation [9]. Proxy measures of external load such as time motion analysis (TMA) suggest that submission grappling is an intermittent activity with effort:pause ratios between 6:1 and 13:1, comprised of effort periods of 85–290 s and pauses of 5–44 s [10, 11]. More recently, however, internal load measured directly by heart rate (HR) during simulated competition revealed physiological responses to submission grappling may be relatively stable [6, 12]. Therefore, whilst specific physical actions in submission grappling may be interrmittant and acyclic, the physiological responses to these actions may not be. As such, methodological limitations inherent within TMA may limit its ability to fully elucidate the contribution of discrete movements or isometric contractions to the overall load of training or competition. This restricts the capacity of TMA to measure the external load of submission grappling. The absence of an accurate external load measurement may cause difficulties in understanding the relationship of external to internal load in this population, leading to suboptimal training prescription and implementation [8].

The use of microelectromechanical systems (MEMS) devices for monitoring external load of athletes is now commonplace across sport [13]. Such devices typically contain both a satellite navigation chip which connects to global navigation satellite systems (GNSS), and also inertial sensors such as accelerometers, gyroscopes and magnetometers [14]. The most common variables to quantify external load using such devices include total distance, high speed distance and metabolic power-based derivatives [15]. Given the minimal influence of distance or ambulatory speed on performance in combat sports, however, the use of positional-based data is limited, with potentially more relevant data provided by the inertial sensors [13]. The most common variable used by practitioners with inertial sensors is the use of accelerometry-based variables such as PlayerLoad [15]. PlayerLoad is an arbitrary unit that is derived from 3-dimensional measures of the instantaneous rate of change of acceleration [16]. Previous research has revealed PlayerLoad demonstrates excellent levels of inter and intra reliability using both a mechanical shaker [17] and human locomotion during treadmill running [16]. Hurst et al. [18] revealed that PlayerLoad demonstrated acceptable levels of reliability for MMA-based movements, including wrestling-based takedowns and ground strikes (CV = 2.4–7.8%, ICC = 0.79–0.98). Body worn accelerometery has since been used for measuring external load in MMA [19, 20], taekwondo [21] and boxing [22, 23]. Recent research has also shown accelerometery to be capable of measuring pacing changes in combat sports inclusive of submission grappling movements [24]. However, there is currently no research supporting the reliability of accelerometry specifically for standing and ground-based transitions and submission manoeuvres common in grappling-based sports [25, 26].

For accelerometery to be used as an acceptable method of external load monitoring in submission grappling inclusive sports, the reliability of this equipment in measuring submission grappling techniques must be determined. Therefore, our primary aim in this study was to determine the intra-unit reliability of PlayerLoad for measuring external load in a series of representative submission grappling movements. In addition, knowledge of between repetition variation in technique and movement as recorded by PlayerLoad would also be required to enable appropriate use of this technology in an applied setting. To this end, a secondary aim was to quantify the variability of PlayerLoad between repetitions of grappling-based movements.

## MATERIALS AND METHODS

### Participants

Inclusion criteria for participants was at least 18 months experience (mean = 6 ± 2 years, range = 2–10 years) of submission grappling, BJJ or MMA training, or holding at least a blue belt in BJJ (male n = 5, female n = 2, age = 31 ± 6 years, stature = 177 ± 13 cm, body mass = 84 ± 18 kg). Participants were recruited following informed consent and institutional ethical approval (Ref: S05-03-21GF059) in keeping with the Declaration of Helsinki.

### Equipment

Participants were requested to wear either a t-shirt or rashguard with shorts or BJJ gi trousers; the wearing of gi jackets or belts was not permitted. Participants were then fitted with a GPS device incorporating a triaxial 100 Hz accelerometer (Optimeye S5, Catapult^®^, Melbourne, Australia), which has previously been shown to have excellent intra-unit reliability [27]. The units were worn in the manufacturers garment, sized so as to ensure a tight fit on the participant, with the unit positioned on the T3-4 vertebrae [28]. Each unit was calibrated on the morning of data collection in keeping with manufacturer instructions. Data collection was filmed and ‘time stamped’ using a tripod-based camcorder with a sampling rate of 60 Hz (HC-V250, Panasonic, Japan) to allow accurate determination of movements on the resultant accelerometery data traces. Data collection using the GPS unit adhered to previously established guidelines for their use in sport [14].

### Protocols

Participants were paired with each other based on body mass and stature. Following a group-based warm up, participants were requested to complete three small jumps on the spot to provide a clear, distinct acceleration marker on the collected data to allow synchronisation with the video recording. The participants were then led through 5 repetitions of each of the submission grappling movements listed in Table 1, with each repetition commencing on the count of one of the authors. Minimum sample size in terms of participants and technique trials was estimated a priori using the guidance provided by Bujang and Baharum [29], in which 3 participants performing 5 trials of a technique would provide β = .9. Following data collection of 7 participants performing 5 trials each, providing a total sample of 35, post hoc power was estimated using ‘ICC.Sample. Size 1.0’ R package in RStudio 1.3.1073 stating a required α < .05 in a two tailed test. Each technique was analysed in turn with β = .98–.99 demonstrating high power. The analysed techniques were selected to represent some of the most fundamental movements from the three main categories of techniques within submission grappling; Passess/Transitions, Sweeps, and Submissions. Technique order was randomised, but all 5 repetitions of each technique were completed before moving onto the next technique. To ensure consistency of technique, participants were shown a video clip of the required technique three times prior to each being completed. Techniques on the video clips were performed by two experienced submission grapplers (both holders of purple belts in BJJ) and can be viewed here: https://osf.io/t6x7q/?view_only=97ecf7d086ce4c40bbbf7c966619fb5f. The techniques of participants were reviewed both during the testing sessions and during data analysis to ensure the correct technique was followed. If an error was spotted during the session, participants were asked to repeat it and if errors were observed during data analysis the action was removed.

**TABLE 1:** Submission grappling techniques included in analyses

Submissions	Transitions	Guard passes	Takedowns
Arm bar from full guard bottom	Butterfly sweep from bottom	Knee slice from full guard top to side control top	Single leg

Arm bar from full mount top	Half guard sweep from bottom	Standing pass from full guard top to side control top	Double leg

Kimura from full guard bottom	Hip bump sweep from full guard bottom		

Guillotine choke from north-south top	Side control top to full mount top		

	North-south top to back (opponent in turtle)		

### Data Treatment and Statistical Analysis

Data were downloaded from the accelerometers and analysed using the manufacturers software (Openfield^TM^, Catapult^®^, Melbourne, Australia). Each participant’s data was individually synchronised with the recorded video of the data collection session. Each movement was determined as the first deviation of the PlayerLoad trace from zero in conjunction with the commencement of movement on the synced video, to the end of the movement according to the video. Data recorded for each movement was accumulated PlayerLoad (PLd_ACC_), calculated from the sum of the magnitude of changes in accelerations in the three cardinal planes (Bredt et al. 2020) designed to measure overall external load (AU). PLd_ACC_ is automatically calculated by the Optimeye units using equation (1) where a_y_ = anterior-posterior changes in acceleration, a_x_ = medial-lateral changes in acceleration, a_z_ = vertical changes in acceleration and t = time [27]:

$$\text{PlayerLoad}=\sqrt{\frac{{\left(a_{y(t)}-a_{y(t-1)}\right)}^{2}+{\left(a_{x(t)}-a_{x(t-1)}\right)}^{2}+{\left(a_{z(t)}-a_{z(t-1)}\right)}^{2}}{100}}$$ (1)

Also recorded was PlayerLoad per minute (PLd_ACC_∙min^-1^), which indicates relative external load (AU). The intra-unit reliability of the units in recording external load of submission grappling movements was determined using two way mixed effect intraclass correlation coefficient (ICC_(3,1)_) using an minimum acceptance threshold ≥ 0.70 [30, 31], ≥ 0.75 classified as ‘good’ and ≥ 0.90 being ‘excellent’ [31] and is presented as point estimate and 95% confidence intervals. Significance was accepted at α < .05. The mean within participant, between repetition variation in PLd_ACC_ and PLd_ACC_∙min^-1^ was calculated using coefficient of variation (CV) with 95% confidence intervals (95%CI). Owing to the highly open skill nature of grappling movements and techniques, a high variability of movement was predicted. In such cases there is likely to be high dispersion of SD around the mean, with a relatively high CV as a result. As such it is recommended to analyse CV within the context of the variable being measured and the action being performed [32]. Therefore, whilst CV ≤ 10% was determined as being good, CV ≤ 15% was deemed acceptable [33]. All statistical analyses were completed using SPSS 26 (IBM, USA).

## RESULTS

The results for PLd_ACC_ reliability can be viewed in Table 2, whilst the reliability for PLd_ACC_∙min^-1^ are in Table 3. All techniques were found to have high ICC_(3,1)_.

**TABLE 2:** Accumulated PlayerLoad (PLd_ACC_) reliability results for all movements.

Technique	ICC_(3,1)_	Upper Bound	Lower Bound
**Submissions**			
Arm bar bottom	0.89	0.98	0.65
Arm bar top	0.95	0.99	0.84
Kimura	0.94	0.99	0.80
North-south guillotine	0.90	0.98	0.71

**Transitions**			
Butterfly sweep	0.93	0.99	0.80
Half guard sweep	0.90	0.98	0.68
Hip bump sweep	0.98	0.99	0.93
Mount from side control	0.94	0.99	0.81
North-south turtle to back	0.78	0.96	0.37

**Guard Passes**			
Knee-slice pass	0.95	0.99	0.83
Standing pass	0.96	0.99	0.88

**Takedowns**			
Single leg	0.92	0.99	0.79
Double leg	0.91	0.98	0.72

*Nb. All ICC α < .05; TD = takedown; ICC = intraclass correlation coefficient*

**TABLE 3:** Accumulated PlayerLoad per minute (PLd_ACC_ ∙ min^-1^) reliability results for all movements.

Technique	ICC_(3,1)_	Upper Bound	Lower Bound
**Submissions**			
Arm bar bottom	0.89	0.98	0.65
Arm bar top	0.95	0.99	0.84
Kimura	0.94	0.99	0.80
North-south guillotine	0.90	0.98	0.71

**Transitions**			
Butterfly sweep	0.93	0.99	0.80
Half guard sweep	0.90	0.98	0.68
Hip bump sweep	0.98	0.99	0.93
Mount from side control	0.94	0.99	0.81
North-south turtle to back	0.78	0.96	0.37

**Guard Passes**			
Knee-slice pass	0.95	0.99	0.83
Standing pass	0.96	0.99	0.88

**Takedowns**			
Single leg	0.92	0.99	0.79
Double leg	0.91	0.98	0.72

*Nb. All ICC α < .05; TD = takedown; ICC = intraclass correlation coefficient*

### Unit Reliability Submissions

PLd_ACC_ ICC_(3,1)_ for all submission movements were high (> 0.89). However, the lower bound for arm bar bottom fell marginally below the minimum threshold. Similarly, ICC_(3,1)_ for PLd_ACC_∙min^-1^ were all high (> 0.84) and were statistically significant, with only the lower bound for the north-south guillotine falling below the a priori threshold (0.55).

### Transitions

PLd_ACC_ for all transitions had statistically significant ICC_(3,1)_ > 0.70, with only north-south turtle to back demonstrating a lower bound < 0.70. In addition, the PLd_ACC_∙min^-1^ all had very high ICC_(3,1)_ (> 0.83). All movements had PL_ACC_∙min^-1^ ICC_(3,1)_ lower bounds above the minimum threshold except for the transition from side control to the mount position (0.52).

### Guard Passes

The standing and knee-slice passes had very high reliability for PLd_ACC_ (≥ 0.95) with lower bounds also above the minimum threshold. Further, the same pattern of reliability was seen for both actions for PLd_ACC_∙min^-1^ (≥ 0.96).

### Takedowns

Both single and double leg takedowns also demonstrated high reliability for PLd_ACC_ and had lower bounds above the minimum threshold of 0.70. ICC_(3,1)_ for PLd_ACC_∙min^-1^ for both actions were also very high with lower bounds > 0.70.

### Between Repetition Movement Variation

Mean between repetition CV was above the good a priori threshold of 10% for PLd_ACC_ and PLd_ACC_∙min^-1^ for all submissions, except for PLd_ACC_ during arm bar top (9[8–11]%), which was the only technique to fall under the acceptable threshold. All transition movements also demonstrated CV > 10%. Three transition techniques had acceptable CV for PLd_ACC_ (13–15%), while all but one was acceptable for PLd_ACC_∙min^-1^ (13–14%). Both guard passes exhibited very similar acceptable CV’s for PLd_ACC_ (15%) and PLd_ACC_∙min^-1^ (14%). The PLd_ACC_∙min^-1^ single leg takedown was the only takedown that had an acceptable CV (14[11–18]%). The lower boundary of CV 95%CI for all techniques fell within the acceptable threshold. Individual participants displayed a wide range of CVs for both PLd_ACC_ and PLd_ACC_∙min^-1^ in each technique measured, as shown in Tables 4 and 5.

**TABLE 4:** Between repetition variation in accumulated PlayerLoad^TM^ (PLd_ACC_) for each participant and the cohort overall

	Submissions				Transitions					Guard Passes		Takedowns	

Participant	Arm bar bottom	Arm bar top	Kimura	North-south guillotine	Butterfly sweep	Half guard sweep	Hip bump sweep	Mount from side control	North-south turtle to back	Knee slice pass	Standing pass	Single leg	Double leg
**1**	8%	13%	12%	15%	16%	16%	11%	18%	23%	13%	7%	22%	38%
**2**	8%	9%	11%	15%	24%	8%	10%	14%	10%	12%	9%	16%	10%
**3**	27%	7%	20%	14%	15%	12%	8%	20%	16%	16%	12%	23%	12%
**4**	14%	8%	9%	22%	18%	15%	13%	15%	7%	13%	22%	13%	22%
**5**	20%	11%	25%	11%	11%	29%	16%	16%	11%	9%	15%	15%	21%
**6**	37%	12%	10%	25%	7%	12%	18%	16%	15%	14%	17%	8%	26%
**7**	25%	10%	25%	24%	10%	**	15%	19%	17%	31%	22%	28%	25%

**Cohort (95% CI)**	17 (13–21)%	11 (7–16)%	16 (10–22)%	19 (14–24)%	13 (9–17)%	14 (7–23)%	13 (10–17)%	14 (8–20)%	17 (11–22)%	14 (12–17)%	14 (10–18)%	14 (11–17)%	17 (10–24)%

*Nb. CV = coefficient of variation; 95%CI = 95% confidence interval;*

** = *unit error resulted in no data*

**TABLE 5:** Between repetition variation in PlayerLoad^TM^ per minute (PLd_ACC_∙min^-1^) for each participant and the cohort overall

	Submissions				Transitions					Guard Passes		Takedowns	

Participant	Arm bar bottom	Arm bar top	Kimura	North-south guillotine	Butterfly sweep	Half guard sweep	Hip bump sweep	Mount from side control	North-south turtle to back	Knee slice pass	Standing pass	Single leg	Double leg
**1**	17%	16%	11%	10%	16%	5%	6%	10%	20%	15%	10%	14%	35%
**2**	7%	11%	8%	23%	16%	11%	15%	15%	7%	13%	9%	13%	18%
**3**	17%	6%	16%	20%	19%	14%	10%	3%	16%	18%	14%	19%	12%
**4**	15%	9%	17%	29%	10%	11%	19%	8%	6%	17%	17%	6%	12%
**5**	19%	7%	24%	11%	11%	35%	17%	24%	13%	8%	17%	15%	20%
**6**	26%	8%	8%	26%	13%	15%	17%	12%	21%	18%	8%	15%	13%
**7**	19%	23%	29%	15%	3%	**	10%	24%	11%	15%	23%	16%	9%

**Cohort (95% CI)**	20 (12–28)%	9 (8–11)%	16 (11–22)%	18 (14–22)%	15 (10–19)%	16 (10–21)%	13 (10–16)%	17 (15–18)%	22 (12–31)%	15 (10–21)%	15 (11–19)%	18 (13–23)%	22 (15–29)%

*Nb. CV = coefficient of variation; 95%CI = 95% confidence interval;*

** = *unit error resulted in no data*

## DISCUSSION

Our aim in this study was to investigate the reliability of the Catapult^®^ PlayerLoad metric in measuring the external load of submission grappling techniques. Our results show that the PLd_ACC_ and PLd_ACC_∙min^-1^ metrics display excellent intra-unit reliability across most techniques, but demonstrate high between repetition variability in measuring individual participant movement. This suggests that whilst PLd may not be capable of differentiating changes in external load of individual techniques between repetitions, it may be effective at quantifying the overall external load of full training sessions within an individual athlete. These results are similar to reliability data from MMA movements which also found high intra-unit reliability but lower inter-unit reliability [18]. Where these studies differ is that in MMA movements the CV = 2.4–7.8%, whilst all techniques in our current study were found to exceed this range (CV = 9–22%).

In viewing our data, there is little difference in ICC_(3,1)_ of PLd_ACC_ or PLd_ACC_∙min^-1^ between the different categories of techniques observed with all techniques demonstrating high to excellent reliability. This indicates that PlayerLoad may be a reliable tool to quantify the external load of grappling-based training sessions. In order to support this proposition, further studies should be completed comparing PlayerLoad across multiple sessions of differing planned intensities to determine this variable’s capability of distinguishing between sessions of higher and lower loads. In addition, within session PlayerLoad should be compared to internal load measurements such as HR or rating of perceived exertion (RPE) to determine a potential dose-response relationship to enable coaches to monitor the load-fatigue-recovery process [8].

Our data do, however, demonstrate high between repetition movement variance as measured by PlayerLoad. A potential explanation for the differences seen in the present study compared to data from the MMA study, may be the nature of the movements being performed. Hurst et al. [18] mostly performed strikes onto a stationary heavy bag. This would allow the participants to move without having to make changes in movement or technique in response to external influence. In addition, the performance of single strikes has fewer stages to each technique [34], and therefore a consistent movement pattern is more likely within individuals. In the current study, and in submission grappling in general, this is not always the case. Participants sometimes complete their movements in response to the movement of their opponent, or attempt to initiate a movement by their opponent to facilitate their own technique. Though participants were instructed not to resist any of the techniques being performed, any slight changes in their balance, mass distribution or subconscious movement would in turn influence the movement of the participant being measured. Moreover, the combination of multiple stages within each grappling technique provides greater chance of measurement variation due to a culmination of small changes within each stage of a technique. The high CVs for each technique is evidence of such small changes within the individuals technique leading to high variation in the measured load of that movement. This was also seen in the takedowns in the aforementioned MMA study [18] having lower bound values < 0.70, which were the only movements where the participants in that instance interacted with another person physically.

Similarly, many movements in submission grappling do not involve changes in acceleration of the torso. For example, isometric gripping or holding actions may not be registered by the device at all. The same may be said for movements in which a participant in a prone position pushes or applies force onto their opponent with their legs only. Neither of these movements would necessarily result in movement of the torso but would still add to the actual load experienced. Previous work has highlighted that microsensors may not be able to capture certain sport specific actions, such as tackling or the scrum in rugby, the latter of which shares some of the high-force low movement actions present in grappling sports [35]. In each of these scenarios, it may be the case that accelerometery would record high, but not low, impulse movements. Given the inherent variation in discrete aspects of a repeated gross skill [36], it is possible that participants were making subconscious alterations to the impulse of the movement being performed on each repetition. If accelerometery is sensitive to changes in movement or technique along this spectrum this may be revealed in a relatively high CV. Therefore, when considering the use of PlayerLoad in a live training or competition setting, increased movement and resistance from the opponent is likely to cause greater between repetition variation in accelerometer measured external load.

A second potential cause of a high variance between repetitions may be the regular changing of the torso angle and position during grappling movements and the unavoidable occurrence of the accelerometer coming into contact with the ground or the opponent. These movement artefacts have been highlighted as a potential reason for inaccurate measurements from accelerometers in field settings [37, 38]. This is a limitation of the use of this technology in grappling sports that cannot be overcome without a change in the structure and design of the units, or the harness used to attach the unit to the participant. Despite these issues, the lower bound 95%CI of all techniques for both variables still fell under the acceptable CV threshold, with 12 falling under the good threshold. This may indicate that PLd_ACC_ and PLd_ACC_∙min^-1^ may have greater consistency when measured with participants capable of more consistent technique performance. It also reinforces the requirement of establishing individual minimal detectable changes for monitoring changes in performance or load. This might entail individual monitoring across a number of sessions of matched and differing intensities to determine each participant’s smallest worthwhile change (SWC) or Z scores for PLd_ACC_ and PLd_ACC_∙min^-1^ [39]. Equally, these data indicate that fixed, prescriptive external load thresholds via accelerometery would not be recommended for submission grappling.

PlayerLoad may be used in research to help determine the external load and pacing of simulated and competitive bouts across a range of grappling-based sports, mirroring its use in MMA research [19, 24]. The contribution of isometric contractions or opponent mass to external load, however, cannot be measured by PlayerLoad. Both of these aspects are likely to be important determinants of load [40]. Equally, there are currently no studies validating PlayerLoad against internal load or gold-standard motion analysis for submission grappling [41]. Due to the continuous physical contact nature of submission grappling, there are inherent difficulties in the collection of internal markers such as HR. Recent studies though have demonstrated that whilst difficult, using HR monitors in simulated BJJ bouts is possible with minor adaptations [12]. Research application of HR telemetry integrated into the GPS harness may make collection of HR data in the field easier and provide a valuable combination of internal and external load measures [42]. Further, the future development of sport-specific algorithms, which have the capacity to detect specific movements [43, 44], would aid in understanding the relationship of internal and external load in grappling-based athletes.

## CONCLUSIONS

In conclusion, high ICCs suggests PlayerLoad may be capable of quantifying the overall external load of grappling-based training sessions. Whilst comparison between individuals may be limited due to high variation between individuals and repetitions, it may play a role for monitoring session-to-session changes for an individual athlete. Coaches may use PlayerLoad to measure the external load of their athletes during submission grappling training and set targets or thresholds for monitoring purposes. Researchers may also use PlayerLoad in simulated or actual competition to determine the external loads that participants should be preparing for. However, the incorporation of physiological measurements and individual thresholds may enhance the validity and usefulness of this measurement for quantifying overall training load. Future work should investigate the use of PlayerLoad in measuring the external load of grappling-based training sessions of differing intensities and planned loads.

## Declarations of Interest

There are no conflicts of interest for any of the authors.
